# Herpes Simplex Virus 1 UL37 Protein Tyrosine Residues Conserved among All Alphaherpesviruses Are Required for Interactions with Glycoprotein K, Cytoplasmic Virion Envelopment, and Infectious Virus Production

**DOI:** 10.1128/JVI.01202-16

**Published:** 2016-10-28

**Authors:** Dmitry V. Chouljenko, Nithya Jambunathan, Vladimir N. Chouljenko, Misagh Naderi, Michal Brylinski, John R. Caskey, Konstantin G. Kousoulas

**Affiliations:** a Division of Biotechnology and Molecular Medicine and Department of Pathobiological Sciences, School of Veterinary Medicine, Louisiana State University, Baton Rouge, Louisiana, USA; b Department of Biological Sciences, College of Basic Sciences, Louisiana State University, Baton Rouge, Louisiana, USA; c Center for Computation & Technology, Louisiana State University, Baton Rouge, Louisiana, USA; Northwestern University

## Abstract

The herpes simplex virus 1 (HSV-1) UL37 protein functions in virion envelopment at *trans*-Golgi membranes, as well as in retrograde and anterograde transport of virion capsids. Recently, we reported that UL37 interacts with glycoprotein K (gK) and its interacting partner protein UL20 (N. Jambunathan, D. Chouljenko, P. Desai, A. S. Charles, R. Subramanian, V. N. Chouljenko, and K. G. Kousoulas, J Virol 88:5927–5935, 2014, http://dx.doi.org/10.1128/JVI.00278-14), facilitating cytoplasmic virion envelopment. Alignment of UL37 homologs encoded by alphaherpesviruses revealed the presence of highly conserved residues in the central portion of the UL37 protein. A cadre of nine UL37 site-specific mutations were produced and tested for their ability to inhibit virion envelopment and infectious virus production. Complementation analysis revealed that replacement of tyrosines 474 and 480 with alanine failed to complement the UL37-null virus, while all other mutated UL37 genes complemented the virus efficiently. The recombinant virus DC474-480 constructed with tyrosines 474, 476, 477, and 480 mutated to alanine residues produced a gK-null-like phenotype characterized by the production of very small plaques and accumulation of capsids in the cytoplasm of infected cells. Recombinant viruses having either tyrosine 476 or 477 replaced with alanine produced a wild-type phenotype. Immunoprecipitation assays revealed that replacement of all four tyrosines with alanines substantially reduced the ability of gK to interact with UL37. Alignment of HSV UL37 with the human cytomegalovirus and Epstein-Barr virus UL37 homologs revealed that Y480 was conserved only for alphaherpesviruses. Collectively, these results suggest that the UL37 conserved tyrosine 480 residue plays a crucial role in interactions with gK to facilitate cytoplasmic virion envelopment and infectious virus production.

**IMPORTANCE** The HSV-1 UL37 protein is conserved among all herpesviruses, functions in both retrograde and anterograde transport of virion capsids, and plays critical roles in cytoplasmic virion envelopment by interacting with gK. We show here that UL37 tyrosine residues conserved among all alphaherpesviruses serve critical roles in cytoplasmic virion envelopment and interactions with gK.

## INTRODUCTION

Herpes simplex virus 1 (HSV-1) is the prototypic member of the Alphaherpesvirinae (1). HSV-1 possesses an ∼152-kb double-stranded DNA genome enclosed within an icosahedral capsid composed of 12 pentavalent and 150 hexavalent capsomeres (2). The capsid is coated with a layer of viral proteins called the tegument, which is enclosed by a lipid envelope originating from host cellular membranes that is enriched with viral glycoproteins and other membrane-associated proteins. A variety of interactions between the viral proteins that make up the capsid, tegument, and envelope are responsible for maintaining the structural integrity of mature virions and for shepherding the viral particle through the complex cytoplasmic envelopment process at *trans*-Golgi network (TGN)-derived vesicles and endosomes (3, 4).

The UL37 gene of HSV-1 encodes a large, 1,123-amino-acid (aa; approximately 120-kDa) highly conserved tegument protein that is essential for viral growth in cell culture and that is crucial for viral assembly and secondary envelopment in the cytoplasm (5–7). The amount of UL37 protein (pUL37) incorporated onto mature virions is tightly regulated, since overexpression of pUL37 does not increase the pUL37 amount incorporated into virions (8). pUL37 forms a complex with pUL36, and this interaction is conserved across all three Herpesviridae subfamilies. Interactions between proteins homologous to pUL36 and pUL37 have been documented in the alphaherpesviruses HSV-1, pseudorabies virus (PRV), and varicella-zoster virus (VZV), in the betaherpesvirus human cytomegalovirus (HCMV), and in the gammaherpesvirus Kaposi's sarcoma-associated herpesvirus (KSHV) (9–15). The presence of pUL36 is necessary for the incorporation of pUL37 onto capsids (16). pUL37 is likely added to capsids after pUL36, since pUL36 is still detected on both HSV-1 and PRV capsids in mutants lacking pUL37 (17, 18). Along with pUL36, pUL37 may be involved in the organization of tegument structure. pUL37 attaches to capsid-bound pUL36 at the vertices, together forming thin flexible strands ranging from 15 to 70 nm in length that extend throughout the tegument, possibly providing a scaffold for the rest of the tegument (19). Deletion of either pUL36 or pUL37 prevents the acquisition of appreciable amounts of tegument in the cytoplasm and blocks cytoplasmic envelopment in HSV-1, resulting in cytoplasmic accumulation of unenveloped capsids (18, 20, 21).

The UL37 protein comprises multiple functional domains. Coimmunoprecipitation experiments revealed that pUL37 domains spanning residues 1 to 300 and residues 568 to 1123 are involved in self-association in the absence of its binding partner pUL36 (5). The pUL37 amino terminus contains an alanine-rich region (ARR) spanning residues 44 to 80, a leucine zipper motif covering residues 203 to 224, and a leucine-rich nuclear export signal (NES) encompassing residues 263 to 272 (5, 22). The carboxyl terminus contains a domain spanning residues 1099 to 1104 involved in binding tumor necrosis factor (TNF) receptor-associated factor 6 (TRAF6) to activate NF-κB pathway signaling (5, 23). The C-terminal 578 to 899 aa of pUL37 can interact with a spectraplakin protein called dystonin/BPAG1 that is known as a cytoskeletal cross-linker involved in microtubule stabilization and transport. Viral replication and cytoplasmic capsid mobility during egress from infected cells are impaired in dystonin-depleted cells, suggesting that pUL37 may play a role in capsid trafficking along microtubules (24). The C terminus of pUL37 is also responsible for binding to pUL36 (5, 25). Scanning alanine mutagenesis of pUL37 revealed that residue D631 of pUL37 mediates binding to pUL36. Altering this residue resulted in significantly decreased ability of the virus to replicate, with mutant viral titers approximately 2 logs lower than those of wild-type virus (25). A plasmid encoding the C-terminal portion of pUL37 spanning residues 568 to 1123 that includes the putative pUL36 interaction site partially rescued a UL37-null virus, indicating that the carboxyl terminus of UL37 is particularly important for infectious virus production (5).

The function of the central portion of pUL37 spanning aa 301 to 567 is not well defined. A mutant HSV-1 with a 12-aa protein C (protC) epitope tag inserted in-frame immediately after residue Y480 of pUL37 exhibited a severe defect in cytoplasmic envelopment and surprisingly was partially complemented for replication and spread when grown on cells expressing pUL20 (26). The inserted protC epitope tag may directly disrupt protein-protein interactions mediated by adjacent residues or even trigger a conformational change in the pUL37 protein that might affect binding to other proteins (26). Phosphorylation is a widespread form of posttranslational modification that can affect a multitude of protein functions, including modulation of protein-protein interactions and control of intracellular trafficking (27, 28). Phosphorylation of proteins such as the p53 tumor suppressor has been shown to mediate conformational changes that can affect protein function and regulation (29). Many viral proteins are also phosphorylated, by either viral or cellular kinases. The HSV-1 tegument includes at least three components that are protein kinases, encoded by the UL13, UL23, and US3 genes (6, 7). The UL37 protein is expressed late in the infection cycle and has been reported to be phosphorylated soon after translation of the UL37 gene. Phosphorylation of pUL37 is thought to be performed by a cellular kinase and is not dependent on the presence of any known HSV-1 binding partner, because pUL37 expressed by a recombinant vaccinia virus has also been observed to be phosphorylated (28). It is not known whether UL37 phosphorylation plays an important role in the virus life cycle.

We have previously reported that pUL37 physically interacts with the membrane proteins pUL20 and glycoprotein K (gK), although the exact locations of the relevant gK and pUL37 binding sites remain unknown (26). pUL20 and gK act as modulators of virus-induced fusion and interact with each other, in addition to binding the major HSV-1 fusion protein gB, and pUL20 has also recently been shown to interact with gM (30–34). In addition to their role in fusion, pUL20 and gK are required for secondary envelopment, and their interactions with pUL37 may serve to facilitate the process of cytoplasmic virion envelopment (26, 35–37).

We sought to better define the function of the central portion of HSV-1 pUL37 by performing scanning alanine mutagenesis of highly conserved amino acid residues. Specifically, we targeted residues likely to be exposed on the protein surface as predicted by computational three-dimensional modeling based on the recently published crystal structure of the closely related PRV pUL37 amino terminus (38). Previously, we reported that the mutant DC480 virus engineered to have a protC epitope tag inserted immediately after the Y480 residue exhibited a UL37-null-like defect for virus replication and cytoplasmic virion envelopment (26). In addition, we constructed and characterized the DC447 virus having the 12-aa protC epitope tag inserted 100 bp downstream of the Y447 residue, as well as mutant viruses that carried tyrosine-to-alanine changes within the central portion of the UL37 protein. Collectively, our results show that UL37 tyrosines conserved among alphaherpesviruses and especially Y474 and Y480 are involved in UL37 interactions with gK and play crucial roles in cytoplasmic virion envelopment.

## MATERIALS AND METHODS

### Cells and viruses.

African green monkey kidney (Vero) cells were obtained from the American Type Culture Collection (Rockville, MD). The Vero cell-based UL37-complementing cell line BD45 was a gift from Prashant Desai (Johns Hopkins University, Baltimore, MD). All cells were maintained in Dulbecco's modified Eagle's medium (Gibco-BRL, Grand Island, NY) supplemented with 10% fetal calf serum and antibiotics.

### Plasmid and mutant virus construction.

All mutations within the UL37 gene sequence were performed using the GeneTailor site-directed mutagenesis system kit from Invitrogen according to the kit manufacturer's instructions. Construction of viral mutants with specific amino acid changes was accomplished in Escherichia coli by using the markerless two-step Red recombination mutagenesis system and synthetic oligonucleotides implemented on the bacterial artificial chromosome (BAC) plasmid pYEbac102 carrying the HSV-1(F) genome (a gift from Y. Kawaguchi, University of Tokyo, Japan). Construction of the HSV-1 mutant virus DC480, which has a 12-amino-acid protein C epitope tag inserted immediately after amino acid 480 of HSV-1 UL37, was described previously (26). The recombinant mutant virus YE102-VC1 (VC1) was modified to express gK and UL20 genes containing V5 and 3×FLAG antigenic epitopes, respectively, and was described previously (39). Double Red recombination was also used to construct the mutant DC474-480, which has the four conserved tyrosines at positions 474, 476, 477, and 480 of HSV-1 UL37 changed to alanine, as well as mutant viruses DC474 and DC476 with single (Y-to-A) amino acid changes at positions 476 and 477. All mutated DNA regions were sequenced to verify the presence of the desired mutations in BACs and the absence of any other spurious mutations on the viral genome.

### Replication kinetics.

Viral growth kinetics was done essentially as we have described earlier. Briefly, viruses were adsorbed on nearly confluent monolayers of Vero or BD45 cell lines into each well of a six-well plate at 4°C for 1 h. The cells were infected at a multiplicity of infection (MOI) of 2. Thereafter, the plates were incubated at 37°C with 5% CO_2_ for 1 h for viral penetration. Any unbound viruses were washed by treatment with low-pH buffer (pH 3.0), and the infection was allowed to proceed for 0, 6, 12, 24, and 48 h postinfection (hpi). The virus titers were averaged, and the standard deviation was calculated for each time point.

### Transfection-infection complementation assay.

Vero cells were transfected with pUL37 constructs (genes cloned into pcDNA 3.3 vector; Invitrogen) UL37-PL262/263-AA, UL37-F294-A, UL37-P408-A, UL37-GF420/421-AA, UL37-Y474-A, UL37-Y480-A, UL37-P519-A, UL37-F620-A, and UL37-P729-A with the noted residues altered to alanine. After 36 h, transfected Vero cells were infected with the UL37-null virus at an MOI of 5. Infected cells were harvested at 24 hpi, and titration was performed using the UL37-complementing cell line BD45. A plasmid expressing pUL20 was used as the negative control. A plasmid expressing wild-type UL37 was used as a positive control. A second positive control consisted of mock transfection, followed by infection with the UL37-null or wild-type HSV-1(F) viruses.

### Immunoprecipitation and immunoblot assays.

Confluent Vero cells in T75 flasks were infected with the double-tagged recombinant virus VC1 (gK-V5 and UL20-FLAG), DC474-480, or F strain virus at an MOI of 2. At 24 hpi, the infected cells were lysed with NP-40 cell lysis buffer (Life Technologies) supplemented with protease inhibitor tablets (Roche). The samples were centrifuged at 13,000 rpm for 10 min at 4°C. The supernatants were then used for immunoprecipitation. The proteins from virus-infected cells were immunoprecipitated using protein G magnetic Dynabeads according to the manufacturer's instructions (Invitrogen). Briefly, the beads were bound to their respective antibodies and left on a nutator for 10 min, followed by the addition of cell lysates. The lysate-bead mixture was kept on the nutator for 10 min at room temperature and subsequently washed three times with phosphate-buffered saline (PBS). The protein was eluted from the magnetic beads in 40 μl of elution buffer and used for immunoblot assays. Sample buffer containing 5% β-mercaptoethanol was added to the protein and heated at 55°C for 15 min. Proteins were resolved in a 4-to-20% SDS-PAGE gel and immobilized on nitrocellulose membranes. Immunoblot assays were carried out using monoclonal mouse anti-FLAG antibody (Sigma-Aldrich, Inc., St. Louis, MO), monoclonal mouse anti-V5 antibody (Invitrogen), mouse monoclonal anti-VP5 antibody, horseradish peroxidase (HRP)-conjugated goat anti-mouse antibodies (Abcam, Inc., Cambridge, MA), polyclonal rabbit anti-UL37 antibody (a gift from Frank J. Jenkins, University of Pittsburgh Cancer Institute), and HRP-conjugated goat anti-rabbit and anti-mouse antibodies (Abcam, Inc., Cambridge, MA).

### Structure modeling of the HSV-1 UL37 protein.

Template-based modeling of the first 570 amino acids of the HSV-1 UL37 protein was conducted by homology modeling with Clustal Omega (40) and Modeler (41). The X-ray crystal structure of PRV UL37 (PDB code 4K70) (38) was used as the modeling template. The amino acid sequence of the HSV-1 UL37 protein (NCBI RefSeq accession no. YP_009137112.1) was used for a multiple-sequence alignment (MSA) between 20 different strains of alphaherpesviruses, including HSV-2 and PRV. The MSA was constructed with the Clustal Omega sequence profile alignment (40) server provided by the Max Planck Institute (http://toolkit.tuebingen.mpg.de/clustalw/). The default options with the modification of 5× HMM were used to run Clustal Omega. After removal of the first 29 residues, the remaining 541 amino acids from the sequence were used to build the homology model with Modeler version 9.15. The HSV-1 UL37 protein model was visualized using molecular visualization package PyMol (PyMOL Molecular Graphics System, version 1.2r3pre; Schrödinger, LLC.). An alignment of HSV-2 UL37 (YP_009137112.1; AKC59563.1) with the beta-herpesvirus HCMV UL37 (Merlin; YP_081505.1) and the gamma-herpesvirus EBV (YP_001129450.1) was performed using the same parameters and software described above for the alphaherpesvirus UL37 proteins.

### Generating sequence logos.

Sequence logos were generated using the Web-based application WebLogo (42) (http://weblogo.berkeley.edu/logo.cgi/) with the default settings. Seven different herpesvirus alignments from the MSA mentioned above were selected to generate the sequence logo. The names of the selected viruses and their accession numbers are as follows: human herpesvirus 1, YP_009137112.1; human herpesvirus 2, AKC59563.1; human herpesvirus 5, YP_081505.1; human herpesvirus 4, YP_001129450.1.

### *In silico* alanine scanning.

Amino acid residues in the UL37 homology model are mutated into alanines one at a time by the AlaScan feature in the protein design package FoldX Suite downloaded in 2016 (http://foldxsuite.crg.eu/); the difference in the Gibbs free energy change (ΔΔ*G*) before and after the performance of each mutation is calculated by the FoldX Suite (43). The numbers are reported as deviations from the median; a positive ΔΔ*G* indicates the importance of an amino acid residue for structure stability and/or relative contribution to protein interaction interface.

### Electron microscopy.

Subconfluent layers of Vero cells in six-well plates were infected with the wild type VC1 and the UL37 mutant DC474-480 at an MOI of 3. After 1 h of infection, the virus was removed, fresh medium was added, and the infection was allowed to continue for 16 h at 37°C and 5% CO_2_. At 16 hpi, cells were fixed with glutaraldehyde fixative and processed for electron microscopy as described previously (26).

## RESULTS

### Delineation of UL37 functional domains.

**FIG 1:**
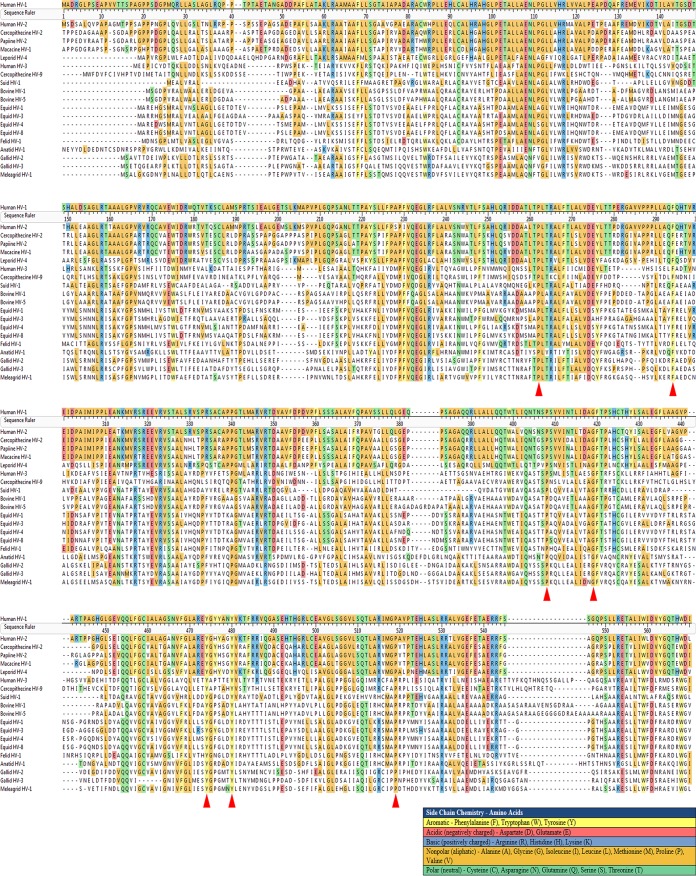
Multiple-sequence alignment of 20 alphaherpesvirus-encoded UL37 proteins. A sequence alignment of the UL37 protein from 20 different alphaherpesviruses encompassing the amino-terminal half of UL37 corresponding to the portion of PRV UL37 with a published crystal structure (38) is shown. Amino acid residues are colored according to their side chain chemistry. Red arrowheads indicate the highly conserved residues that were mutated to alanines.

**FIG 2:**
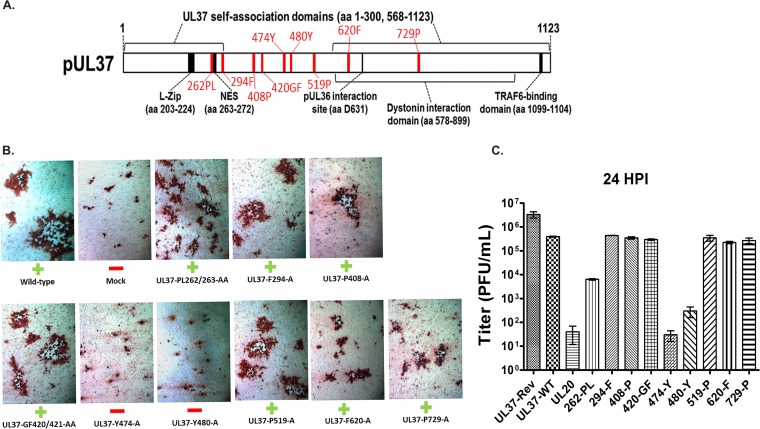
Delineation of UL37 amino acids involved in infectious virus production. (A) Schematic of the UL37 protein depicting known functional domains and the approximate locations of nine single- and double-amino-acid replacements constructed to assess their role in infectious virus production. (B) Images of viral plaques produced in the transfection-infection complementation of the UL37-null virus. Vero cells were transfected with each plasmid. After 36 h, plasmid-transfected cells were infected with UL37-null virus at an MOI of 5 and virus stocks were collected at 24 hpi. Viral plaques were visualized by immunohistochemistry at 48 hpi (see Materials and Methods). The plasmids encoding mutations in the amino acids of UL37 that were able to complement the UL37-null virus are marked with a green plus, and the mutations which were not able to complement are marked by a red minus. (C) Viral titers obtained in the transfection-infection complementation assay. Titers of virus stocks collected at 24 hpi with the UL37-null virus were determined on the BD45 cell line, which expresses the UL37 protein in *trans*. A plasmid expressing the wild-type UL37 protein was used as a positive control. A second positive control consisted of mock transfection, followed by infection with wild-type HSV-1, while a UL20-expressing plasmid was used as a negative control.

To delineate functional domains of the UL37 protein involved in infectious virus production, a cadre of UL37 gene mutations were generated by PCR-assisted mutagenesis targeting residues conserved among alphaherpesviruses. Alignment of a number of UL37 amino acid sequences specified by different alphaherpesviruses revealed that the amino acids proline 262, leucine 263, phenylalanine 294, proline 408, glycine 420, phenyalananine 421, tyrosine 474 and 480, proline 519, phenylananine 620, and proline 729 exhibited a high degree of conservation (Fig. 1). These residues were mutated to alanine residues as single or double mutations in instances where the two conserved amino acids were located adjacent to each other, generating the mutant plasmids UL37-PL262/263-AA (adjacent proline and leucine residues changed to alanines), UL37-F294-A (phenylalanine at amino acid position 294 changed to alanine), UL37-P408-A (proline changed to alanine), UL37-GF420/421-AA (glycine and phenylalanine changed to alanines), UL37-Y474-A and UL37 Y480-A (tyrosines changed to alanines), UL37-P519-A (proline changed to alanine), UL37-F620-A (phenylalanine changed to alanine), and UL37-P729-A (proline changed to alanine). Plasmids expressing the mutant UL37 proteins were tested for their ability to complement a UL37-null virus by transfecting each plasmid into Vero cells, followed by infection with the UL37-null virus grown in the UL37-complementing cell line BD45 (see Materials and Methods). All plasmids complemented the UL37-null virus replication and spread to different extents, except plasmids that specified the UL37-Y474-A and UL37-Y480-A mutations, as evidenced by the appearance of substantially larger viral plaques than those in the mock-transfected cells and the resultant accumulation of infectious virions (Fig. 2A, B, and C).

### Structure modeling of the HSV-1 UL37 protein.

**FIG 3:**
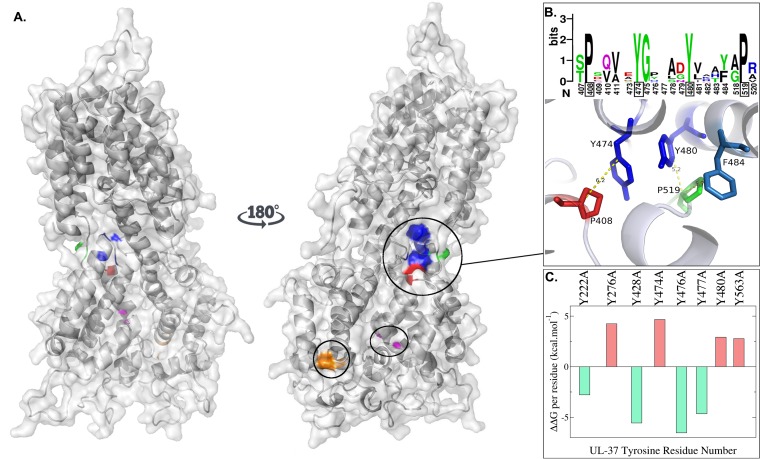
Predicted structure and dynamics of the HSV-1 UL37 protein. (A) Space-filling predictive model of 570 amino acids in the amino terminus of pUL37 in HSV-1 based on the known crystal structure of a corresponding amino-terminal region of PRV UL37. Blue, Y476-Y477 and Y480; orange, P262-L263; red, P408; green, P519; magenta, G419-F420. (B) Sequence logo depicts the relative conservation of amino acids 407 to 411, 473 to 484, and 518 to 520 (top portion of panel B). Approximate distances between the aromatic residues Y474, Y480, and F484 and P408 and P519 (bottom portion of panel B). (C) *In silico* alanine-scanning mutagenesis. The difference between the free Gibbs energy before and after the mutation (ΔΔ*G*) is shown on the *y* axis. Values are reported as the deviation from the median ΔΔ*G* of UL37 tyrosine-to-alanine mutations. Negative values indicate stabilizing mutations (green). Positive values indicate destabilizing mutations (red).

To visualize whether tyrosine residues identified to play important roles in the structure and function of the UL37 protein are exposed on the UL37 surface, where they would be available to participate in protein-protein interactions, we generated the three-dimensional structure of the HSV-1 UL37 protein via homology modeling based on the X-ray crystal structure of the PRV UL37 protein (see Materials and Methods). The Y474 and Y480 residues are more conserved than Y476 and Y477, suggesting that they are important for UL37 structural stability and function. A closer examination of the UL37 structure reveals that Y474 and Y480 are located in a pocket-like structure together with three other highly conserved residues, P408, F484, and P519, forming a helix-turn-helix motif. Further, the presence of two highly conserved prolines, P408 and P519, within 6 Å of these aromatic residues suggests the possibility of C-H-π (CH/π) interactions (π refers to the hybridization of the aromatic carbons, which is also known as a π system, and CH/π is a form of cation–π interactions where a positive atom [in this case hydrogen from a proline residue] interacts with the negative face of the aromatic ring). Together, these putative interactions may have a role in the stability of the protein, especially in preserving the pocket-like structure which might be important in the interaction of UL37 with other proteins (Fig. 3B).

To further determine the role of each tyrosine on the structure of UL37, the relative importance of the conserved tyrosines at positions 474, 476, 477, and 480 on the structural stability of the UL37 proteins was analyzed via computational alanine scanning. In this analysis the differences in the calculated free Gibbs energy change (ΔΔ*G*) were measured between the wild-type and each of the mutated proteins in order to quantify the effect of tyrosine replacement with alanine (see Materials and Methods). These differential energy calculations revealed that the Y276-A, Y474-A, Y480-A, and Y563-A mutations resulted in positive ΔΔ*G* values, suggesting that they are important for the stability of the UL37 structure and/or the ability of the UL37 protein to interact with other proteins (Fig. 3C).

### Construction and characterization of UL37 mutant viruses.

**FIG 4:**
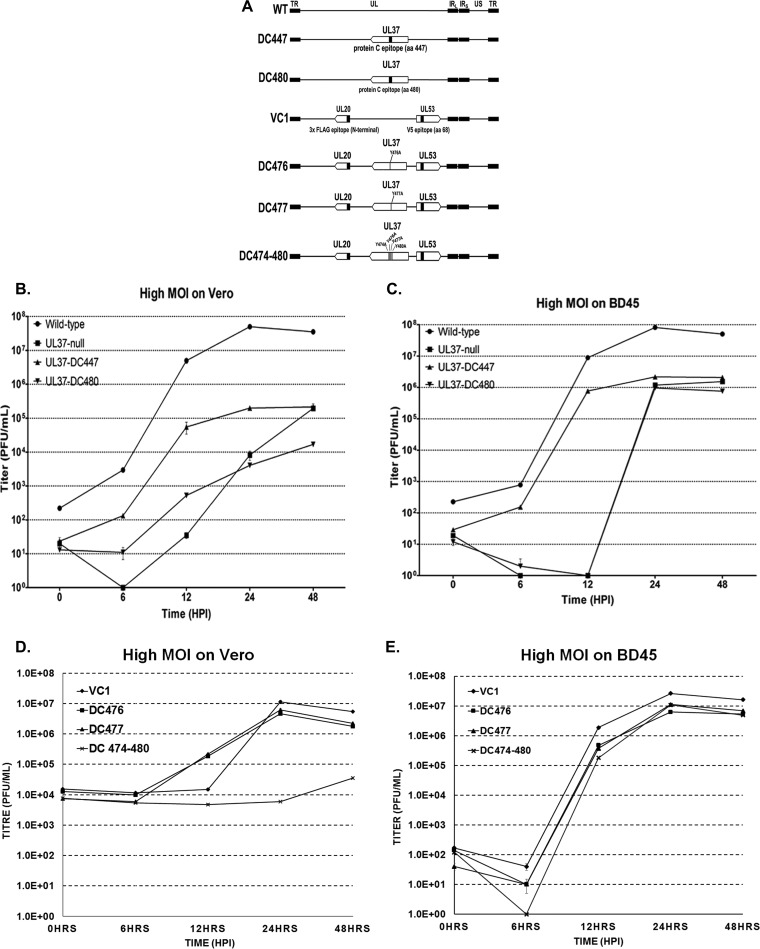
Construction and characterization of UL37 mutant viruses. (A) Schematic representation of the UL37 mutant viruses constructed on the viral genomic background via double-Red mutagenesis on the HSV-1(F) and VC1 genomes cloned as a bacterial artificial chromosome. The approximate locations of the constructed mutations are shown. (B to E) Replication kinetics of wild-type and selected mutant viruses on Vero and BD45 cells. Viruses were adsorbed on nearly confluent monolayers of Vero or BD45 cells. Cells were infected with each virus at an MOI of 2. Virus stocks were collected at the indicated times, and titers were determined on both Vero and BD45 cells. Virus titers from three independent cultures were averaged, and the standard deviation was calculated for each time point.

The mutant virus DC480, which contains a protein C (protC) epitope tag inserted into the HSV-1 UL37 gene immediately after the codon for amino acid 480 in the HSV-1(F) genetic background, has been described previously (26). To further assess the function of this central portion of the UL37 protein in virus replication, we constructed the mutant virus DC447 by inserting the protC epitope tag immediately after amino acid codon 447. In addition, we constructed mutant viruses DC476, DC477, and DC474-480, in which tyrosines at individual positions 476 and 477 or at all four positions (474, 476, 477, 480) were changed to alanine residues, respectively. These mutations were constructed in the VC1 genomic background expressing gK tagged with a V5 epitope and UL20 tagged with a FLAG epitope (Fig. 4A).

The DC480 mutant failed to replicate, producing viral titers similar to those of the UL37-null virus, which is characterized by infectious virus production that is approximately 3 logs lower than that of the parental HSV-1(F) virus, as we have reported previously (26). In contrast, the DC447 virus replicated substantially better than the DC480 and UL37-null viruses; however, final infectious virus production was more than 1 log lower than that of the parental HSV-1(F) virus (Fig. 4B). The DC476 and DC477 viruses, having each tyrosine (tyrosines 476 and 477) mutated to alanine, produced titers similar to that of the VC1 virus, and the DC474-480 mutant, having all four tyrosines (tyrosines 474, 476, 477, and 480) mutated to alanines, produced viral titers similar to those of the UL37-null virus (Fig. 4D). All mutant viruses that exhibited a replication defect in Vero cells were efficiently complemented when grown in the BD45 cell line that expresses the UL37 gene in *trans* (Fig. 4C and E).

**FIG 5:**
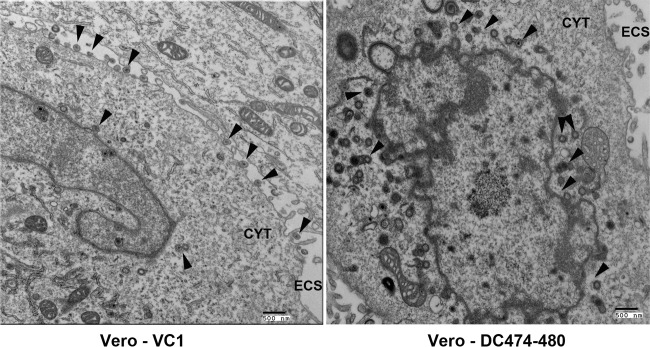
Electron micrographs of Vero cells infected with VC1 or DC474-480 viruses. Vero cells were infected with either VC1 or DC474-480 viruses at an MOI of 5 and visualized by electron microscopy after 24 hpi. Arrowheads indicate the presence of enveloped virions in the periphery of VC1-infected cells and capsids within the cytoplasm of DC474-480-infected cells. The cytoplasm (CYT) and extracellular space (ECS) are indicated.

We have previously reported that the DC480 virus produced a UL37-null phenotype that resulted in the accumulation of capsids in the cytoplasm of infected Vero cells in a manner reminiscent to the gK-null and UL20-null virions, indicating a defect in cytoplasmic virion envelopment (26). Transmission electron microscopic examination of Vero cells infected with the DC474-480 virus revealed a similar defect, characterized by the accumulation of viral capsids in the cytoplasm and the absence of infectious virions in the periphery of infected Vero cells, in comparison to the parental VC1 virus (Fig. 5).

### Role of conserved UL37 tyrosines in UL37-gK interactions.

**FIG 6:**
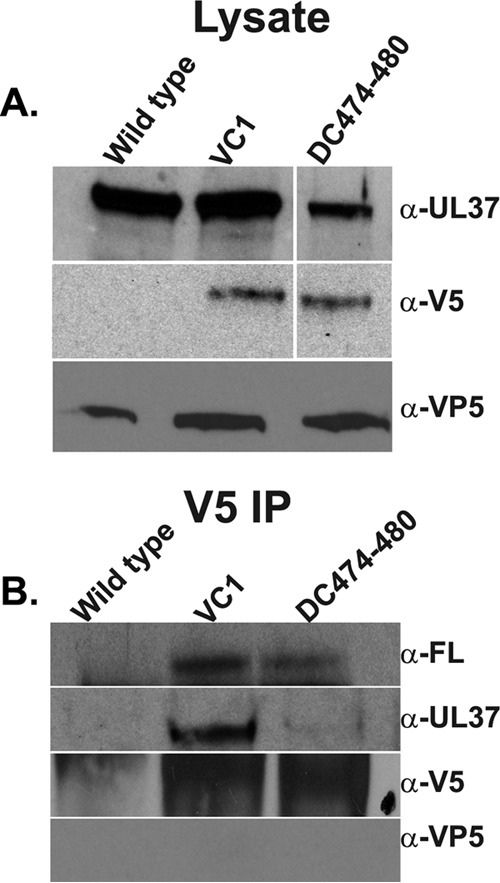
Effect of the DC474-480 mutations on UL37 interactions with gK. (A) Lysates of Vero cells infected with either wild-type HSV-1(F), VC1, or DC474-480 viruses were collected at 24 hpi, electrophoretically separated, blotted onto nitrocellulose membranes, and reacted with either anti-V5 (gK), anti-UL37, or anti-VP5 antibodies. The lanes in the blot for anti-UL37 and anti-V5 panels were spliced from different sections of the same blot, identified by the white line separating the lanes. (B) Lysates prepared as described for panel A were immunoprecipitated with the anti-V5 (gK) antibody. Electrophoretically separated proteins of the immunoprecipitates were blotted onto nitrocellulose membranes, and the presence of UL37 gK, UL20, or UL37 protein was detected by reacting the blots with either anti-V5, anti-FLAG, or anti-UL37 antibodies, respectively. No VP5 was detected when the immunoprecipitate was probed with anti-VP5 antibody (negative control).

We have shown previously that the gK/UL20 protein complex interacts with UL37 (26). Coimmunoprecipitation experiments were performed to evaluate whether the combined Y474-A, Y476-A, Y477-A, and Y480-A mutations specified by the DC474-480 mutant virus affect the ability of UL37 to interact with gK. The VC1 virus was used as a positive control and the HSV-1(F) virus was used as a negative control (the VC1 virus specifies gK and UL20 tagged with V5 and FLAG epitopes, respectively, while the F strain is not epitope tagged). The UL37 protein was detected in all virus-infected lysates probed with anti-UL37 polyclonal rabbit antibody, although the amount of protein detected in the DC474-480 cell lysates was less than in the VC1 or the HSV-1(F) cell lysates. All cell lysates contained similar amounts of the major capsid protein VP5, indicating a similar overall viral protein content across samples. Immunoblot probing of anti-gK (V5) immunoprecipitates revealed the presence of UL20, gK, and pUL37 when probed with anti-UL20 (FLAG), anti-gK (V5), and anti-UL37 antibodies, respectively, for all viruses except HSV-1(F), which does not have gK or UL20 proteins tagged with the V5 or FLAG epitope tags. Importantly, anti-gK (V5) immunoprecipitates contained very small amounts of UL37 appearing as very faintly labeled protein species for DC474-480 in comparison to the VC1 virus, while similar amounts of UL20 protein were immunoprecipitated from the same samples. The gK (V5) immunoprecipitates did not contain the VP5 protein (negative control) (Fig. 6).

## DISCUSSION

The HSV-1 UL37 protein exhibits a high level of conservation among all alphaherpesviruses. Previously, we and others have shown that the UL37 protein plays a critical role in cytoplasmic virion envelopment and egress of virions from infected cells and that the UL37 protein interacts with glycoprotein K (gK) (26). Herein, we show that specific tyrosine residues conserved among alphaherpesviruses, which are predicted to be exposed on the UL37 surface, are critically involved in interaction with gK, cytoplasmic envelopment, egress, and infectious virus production.

Both pUL37 and the UL20/gK proteins are conserved within the neurotropic Alphaherpesvirinae subfamily, reflecting their important roles in the viral life cycle (44). Initial alignment of a number of UL37 homologs showed the presence of highly conserved residues scattered throughout the pUL37 open reading frame (ORF) (25). Alignment of UL37 homologs from 20 different alphaherpesviruses confirmed the conservation of a number of amino acid residues, including tyrosines (Y474 and Y480). Transfection-complementation of a UL37-null virus by the use of a cadre of plasmids carrying specific mutations in UL37 showed that both Y474 and Y480 serve important roles in the pUL37 structure and function(s). This hypothesis was further supported by the UL37 predicted three-dimensional structure, which revealed that Y474 and Y480 were exposed on the UL37 surface and embedded within a “clef” domain reminiscent of a protein binding site.

Additional evidence that tyrosines at positions 474 and 480 were particularly important in UL37 functions was obtained from the characterization of the Y474-Y480 mutant virus in which all four tyrosines at positions 474, 476, 477, and 480 were mutated to alanines. This virus produced a UL37-null phenotype characterized by the accumulation of capsids in the cytoplasm of infected cells and the inability to produce infectious virions. Since the DC476 and DC477 mutant viruses having only the Y476 or Y477 residue changed to alanine replicated fairly efficiently, it can be concluded that Y474 and Y480 play crucial roles in the structure and function of the UL37 protein. This conclusion is supported by the inability of plasmids having either the Y474-A or Y480-A mutation to complement UL37-null virus replication, suggesting that each of these two tyrosine residues plays an important role in the pUL37 structure and function. It was previously reported that the UL37 protein is phosphorylated (28). In contrast to these findings, multiple experiments failed to detect significant UL37 labeling with ^32^P in infected Vero cells (not shown), indicating that phosphorylation of both the Y474 and Y480 residues does not play an important role in UL37 structure and function.

**FIG 7:**
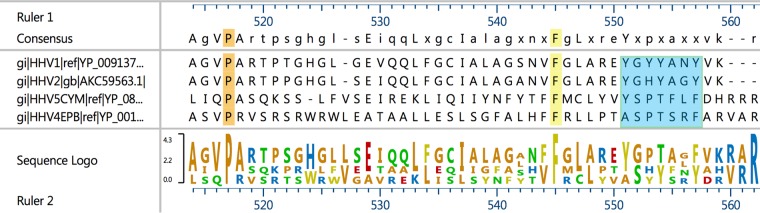
Sequence alignment of alpha-, beta-, and gammaherpesvirus UL37 proteins. Sequence alignment of HSV-1, HSV-2, HCMV, and EBV UL37 proteins was performed as described in Materials and Methods. The region is similar to the aa 470-to-485 codon region of Fig. 1. The consensus sequence logo depicts the relative conservation of each residue. Orange highlights conserved proline, and yellow highlights conserved phenylalanine. The region Y551-Y557 corresponding to Y474-Y480 shown in Fig. 1 is highlighted in blue.

We have shown previously that the UL37 protein interacts with gK and that UL37-null and gK-null viruses produce similar phenotypes characterized by drastic defects in cytoplasmic envelopment and egress (26). Mutagenesis of the four conserved tyrosine residues to alanine revealed that they inhibited interactions of the UL37 protein with gK, suggesting that they are involved in these interactions. The alanine scanning study with FoldX shows that the Y474-A and Y480-A mutations caused a more positive residual free energy (ΔΔ*G*) representing an unfavorable or destabilizing change than the Y476-A and Y477-A mutations. The location of the Y474 and Y480 residues in a pocket-like helix-turn-helix motif may play an important role in preserving the structure of this putative binding/interaction site, especially in preserving the geometry of the helix-turn-helix motif, which is in general known to be important for protein-protein interactions (45, 46). Also, it is possible that Y474 and Y480 contribute to the preservation of the three-dimensional structure of the entire UL37 protein. However, the fact that Y480 is conserved only in alphaherpesviruses, not in beta- or gammaherpesviruses (Fig. 7), argues that Y480 is particularly important for interactions with gK. It is worth noting that the precision of the energy calculation by alanine scanning methods can be assessed using experimental free-energy measurements. In the current study, we have focused on the relative changes of Δ*G* rather than the absolute values in order to reduce any possible bias from energy calculations and to eliminate the need to examine the precision of such methods.

Apparently, the drastic reduction in gK interactions with pUL37 did not affect the interactions between the UL20 protein and pUL37. We have reported previously that TGN localization of gK requires coexpression of the UL20 protein. However, UL20 assumes a cytoplasmic distribution in the absence of gK, while gK is trapped into rough endoplasmic reticulum (RER) membranes (33). Thus, it is conceivable that UL20 protein not bound to gK may interact with pUL37 independently of gK to facilitate cytoplasmic virion envelopment.

HSV-1 assembles in infected cells through a sequentially ordered process depending on precise protein-protein interactions that regulate intracellular transport and virion egress, as well as the amount and nature of each structural protein, which is ultimately incorporated within the mature virion. It has been reported that the UL36/UL37 protein complex can be transported to TGN membranes, where cytoplasmic envelopment occurs in the absence of capsid formation (47). This result suggests that the UL36/UL37 complex may anchor to TGN membranes via binding to cytoplasmic domains of gK and UL20 proteins. Overall, our present and previous results indicate that physical interactions between the gK/UL20 protein complex and the UL37 protein serve important functions in cytoplasmic virion envelopment and infectious virus production.

We report here for the first time the HSV-1 UL37 structure model based on the overall conserved nature of the UL37 proteins encoded by alphaherpesviruses and the available X-ray structure of the PRV UL37. In addition, we utilized *in silico* alanine mutagenesis to analyze the relative importance of different amino acid residues in UL37 structure and function, providing significant complementary evidence of the relative role of conserved amino acid residues in UL37 structure and function. This computational approach should be useful to explore the structure and function of other viral and cellular proteins.
